# Biological Activity of Novel Pyrrole Derivatives as Antioxidant Agents Against 6-OHDA Induced Neurotoxicity in PC12 Cells

**DOI:** 10.5812/ijpr-140450

**Published:** 2023-12-24

**Authors:** Hanieh Javid, Ebrahim Saeedian Moghadam, Maryam Farahmandfar, Mahboubeh Manouchehrabadi, Mohsen Amini, Mona Salimi, Anahita Torkaman-Boutorabi

**Affiliations:** 1 Department of Neuroscience and Addiction Studies, School of Advanced Technologies in Medicine, Tehran University of Medical Sciences, Tehran, Iran; 2 Department of Medicinal Chemistry, Faculty of Pharmacy, Tehran University of Medical Sciences, Tehran, Iran; 3 Drug Design & Development Research Center, Tehran University of Medical Sciences, Tehran, Iran; 4 Department of Physiology and Pharmacology, Pasteur Institute of Iran, Tehran, Iran; 5 Research Center for Cognitive and Behavioral Sciences, Tehran University of Medical Sciences, Tehran, Iran

**Keywords:** COX-2, Neuroprotection, 6-OHDA, Parkinson’s Disease, PC12, PGE2, Pyrrole Derivates

## Abstract

**Background:**

Neuroinflammation and oxidative stress are critical factors involved in the pathogenesis of Parkinson's disease (PD), the second most common progressive neurodegenerative disease. Additionally, lipid peroxidation end products contribute to inflammatory responses by activating pro-inflammatory genes. Lipid peroxidation occurs as a result of either the overproduction of intracellular reactive oxygen species (ROS) or the reaction of cyclooxygenases (COXs).

**Objectives:**

In this study, we examined the role of 1,5-diaryl pyrrole derivatives against the neurotoxic effects of 6-hydroxydopamine (6-OHDA) in a cellular model of PD.

**Methods:**

PC12 cells were pre-treated with compounds 2-(4-chlorophenyl)-5-methyl-1-(4-(trifluoromethoxy)phenyl)-1H-pyrrole (A), 2-(4-chlorophenyl)-1-(4-methoxyphenyl)-5-methyl-1H-pyrrole (B), and 1-(2-chlorophenyl)-2-(4-chlorophenyl)-5-methyl-1H-pyrrole (C), respectively, 24 h before exposure to 6-OHDA. We conducted various assays, including 3-(4,5-dimethylthiazol-2-yl)-2,5-diphenyl-tetrazoliumbromide (MTT), ROS, and lipid peroxidation assays, Hoechst staining, Annexin V/PI, Western blotting analysis and ELISA method, to assess the neuroprotective effects of pyrrole derivatives on 6-OHDA-induced neurotoxicity.

**Results:**

Our results demonstrated that apoptosis induction was inhibited by controlling the lipid peroxidation process in the in vitro model following pre-treatment with compounds A, B, and, somehow, C. Furthermore, compounds A and C likely act by suppressing the COX-2/PGE2 pathway, a mechanism not attributed to compound B.

**Conclusions:**

These findings suggest that the novel synthetic pyrrolic derivatives may be considered promising neuroprotective agents that can potentially prevent the progression of PD.

## 1. Background

Parkinson's disease (PD) is the second most common progressive neurodegenerative disease after Alzheimer's disease, primarily affecting the elderly population. In PD, neurodegeneration occurs in dopaminergic neurons located in the substantia nigra (SN) (1). While its precise etiology remains incompletely understood, several factors have been implicated in exacerbating the degeneration of dopaminergic neurons in the SN. These factors include the accumulation of reactive oxygen species (ROS), lipid peroxidation, and neuroinflammation (2). The buildup of ROS leads to cellular dysfunction, resulting in cell death through DNA damage, inflammation, and protein modification (3). Furthermore, once initiated, lipid peroxidation triggers a chain reaction that propagates to damage more intracellular lipids and proteins.

Various experimental animal models of PD and postmortem findings have highlighted the role of neuroinflammation in the progression of the disease. Neuroinflammation increases the risk of neurodegeneration by upregulating the expression of cyclooxygenase-2 (COX-2). Cyclooxygenases, also known as prostaglandin (PG) H synthase, is the primary enzyme responsible for converting arachidonic acid into PG H2, a key precursor of various PGs (4). Evidence indicates an overexpression of COX-2 in nigrostriatal dopaminergic neurons in both MPTP mice and human PD samples, emphasizing the involvement of COX-2 in neuronal demise (5). Additionally, COX-2 overexpression is associated with an increase in prostaglandin E2 (PGE2). Prostaglandin E2 is known to promote inflammation, and its accumulation has been observed in the SN of postmortem brain samples from PD patients (6). Prostaglandin E2 also contributes to ROS production and activates astrocytes (7). In this context, COX-2 inhibitors have demonstrated a neuroprotective role, reducing 6-hydroxydopamine (6-OHDA)-induced cell death in dopaminergic neurons (4).

The current therapy for PD involves L-DOPA; however, prolonged use of L-DOPA leads to the excessive production of dopamine in the cytosol and the formation of DA quinone, which is closely associated with oxidative stress and inflammation (8). Therefore, the development of novel compounds capable of inhibiting neuronal oxidation and neuroinflammation presents a promising therapeutic strategy to slow the progression and mitigate the severity of PD.

Pyrrole, a valuable nitrogen-containing heterocyclic 5-membered ring, finds applications in a wide range of pharmaceutical products. Various pharmacological studies have reported the remarkable anti-inflammatory and anti-nociceptive activities of different pyrrole derivatives (9, 10). N-heterocycles, particularly the pyrrole motif, serve as attractive scaffolds in medicinal chemistry for the synthesis of novel bioactive compounds with therapeutic potential in areas such as anticancer, antimicrobial, and antiviral treatments (11). Some of these compounds have demonstrated COX-2 selective inhibition and a strong suppressive effect on PGE2 production (12). Consequently, considerable efforts have been directed toward the synthesis of pyrrolic compounds as COX inhibitors for treating inflammatory diseases, with ongoing exploration of their application in neurodegenerative conditions like PD.

## 2. Objectives

In the present study, 3 novel pyrrole derivatives were synthesized and evaluated for their neuroprotective effects by modulating COX-2 expression and PGE2 production.

## 3. Methods

### 3.1. Synthesis

**Figure 1.**
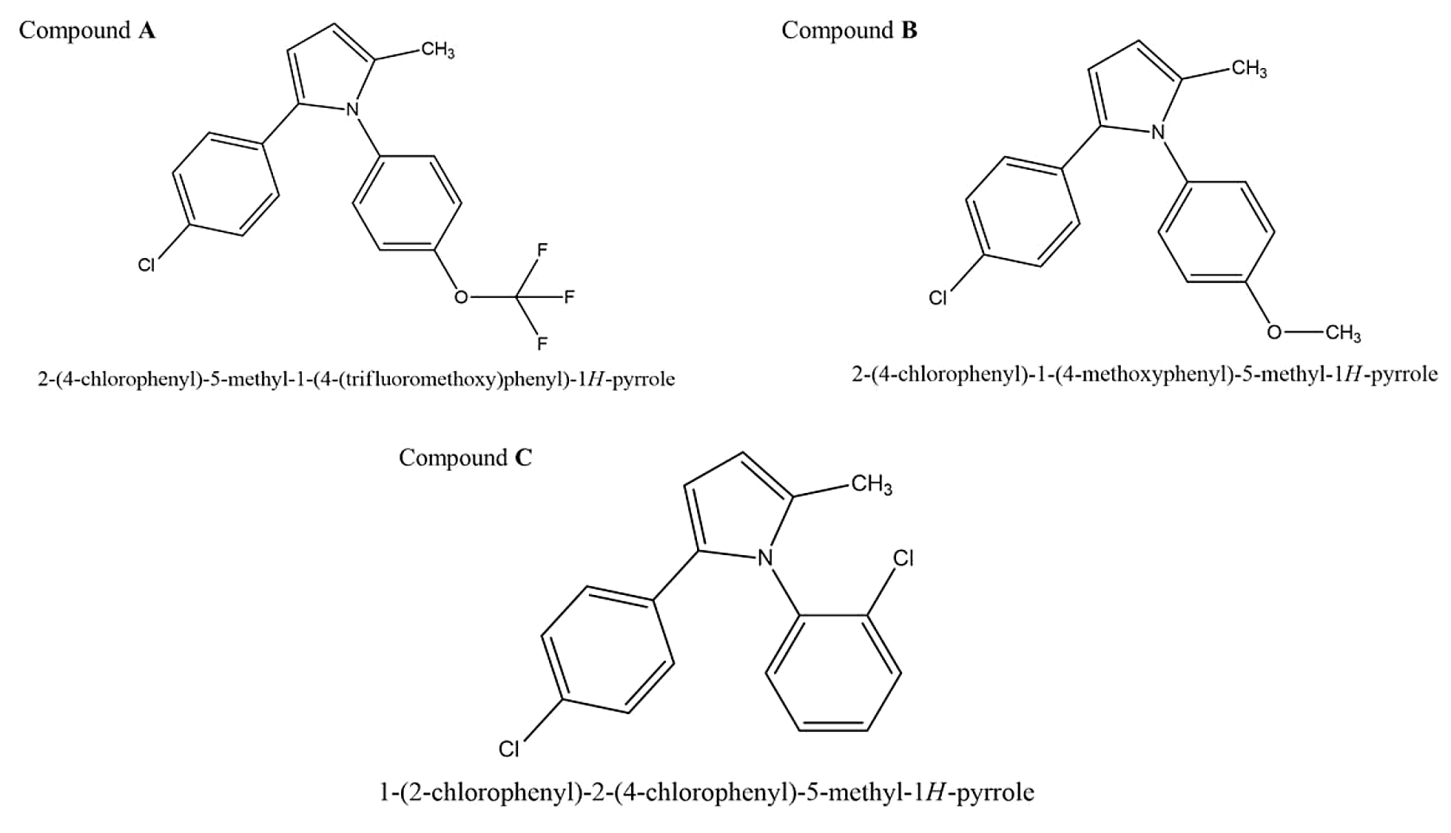
Scheme depicting target compounds A, B, and C.

Compounds A, B, and C were synthesized and characterized as outlined in the supplementary data (Figure 1). 

### 3.2. Cell Culture

**Figure 2.**
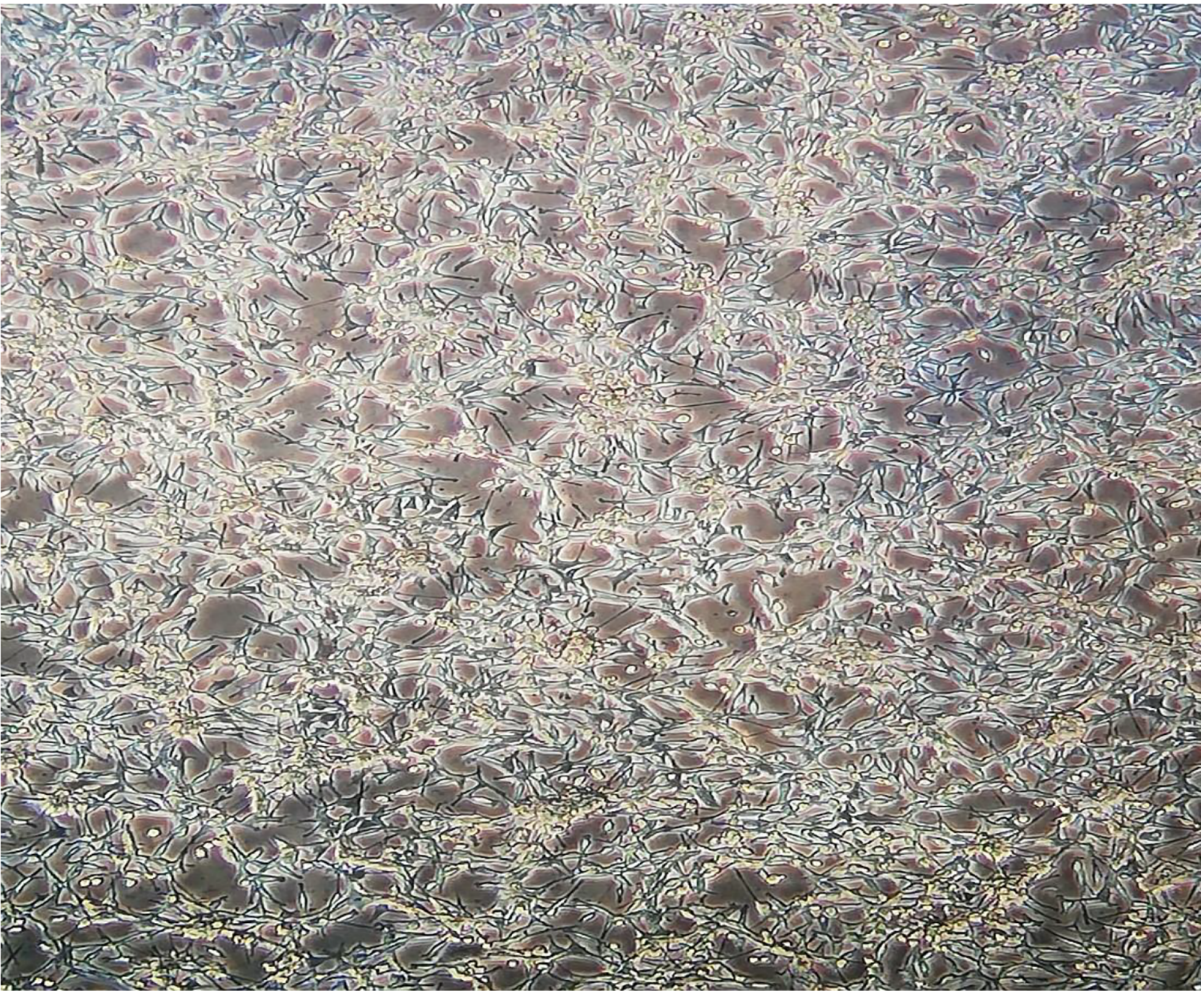
PC12 cell morphology observed under a light microscope (10X magnification).

PC12 cells, a type of catecholamine cells derived from a rat adrenal medulla pheochromocytoma, were procured from the Pasteur Institute of Iran. The cells were cultured in RPMI 1 640 medium (Gibco, USA) supplemented with 10% fetal bovine serum (FBS; Gibco, USA), 100 μg/mL streptomycin, and 100 U/mL penicillin (Gibco, USA). They were maintained in an incubator at 37°C, 5% CO2, and passaged twice a week (Figure 2). 

### 3.3. In Vitro Model and Experimental Design

To determine the optimal concentration of 6-OHDA for inducing a cellular PD model, PC12 cells were exposed to various concentrations (25, 50, 75, 100, 125, 150, and 175 μM) of 6-OHDA from Sigma-Aldrich, a neurotoxin mimicking idiopathic PD pathogenesis in vitro. In order to assess the protective role of the synthetic compounds against 6-OHDA-induced neurotoxicity, PC12 cells were pre-treated with 0.1, 0.5, 1, and 5 μM of pyrrolic compounds. The study groups included: (1) Control group (cells with no treatment); (2) 6-OHDA’s vehicle group (cells treated with DMSO); (3) 6-OHDA groups (cells treated with different concentrations of 6-OHDA for 24 h); (4) compounds A/B/C + 6-OHDA group (cells pre-treated with compounds A or B or C [0.5 μM] for 24 h, then treated with 6-OHDA [100 μM] for another 24 h).

### 3.4. Cell Viability Assay

Quantitative colorimetric 3-(4,5-dimethylthiazol-2-yl) -2,5-diphenyl-tetrazoliumbromide (MTT) assay was employed to determine cell viability. Both untreated and treated cells were incubated with a 5 mg/mL solution of MTT from Sigma-Aldrich, followed by the addition of DMSO to solubilize the formed dark blue formazan crystals. The absorbance of the plate was measured using a microplate reader (BioTek, ELx800, USA) at 570 nm, with background correction performed at 620 nm. Cell viability was calculated as a percentage using the following formula: % Cell viability = 100 × (absorbance of sample/absorbance of control).

### 3.5. Lipid Peroxidation Assay

The thiobarbituric acid (TBA) assay (Merck, Germany) was conducted to determine the extent of lipid oxidative degradation. For this purpose, both untreated and treated cells were harvested using a cell scraper, washed with phosphate-buffered saline (PBS), and centrifuged at 1200×g for 2 min. The color reagent was prepared by dissolving 320 mg of thiobarbituric acid reactive substances (TBARS) in 30 mL of 0.1 M NaOH and 30 mL of 3.5 M diluted acetic acid. Next, 100 μL of 1% sodium dodecyl sulfate (SDS) was added to 100 μL of whole cell lysate and mixed with 4 mL of the TBA reagent to generate colored compounds. The mixture was then boiled for 1 h at 100°C in the dark, and fluorescence intensity was measured at 520 nm excitation and 550 nm emission wavelengths using a BioTek microplate reader (ELx800, USA).

### 3.6. Reactive Oxygen Species Assay

Intracellular ROS levels were determined using a fluorescent probe called 2',7'-dichlorofluorescein diacetate (DCFH2-DA) (13). DCFH2-DA (5 μM) was added to both untreated and treated PC12 cells and incubated in an incubator for 30 min. The cells were then washed, and the fluorescence intensity of dichlorodihydrofluorescein (DCF) was measured at an excitation wavelength of 480 nm and an emission wavelength of 530 nm.

### 3.7. Hoechst Staining

Hoechst 33258 staining was employed to distinguish apoptotic cells from normal cells (14). After fixation, cells were stained with 0.5 μM of Hoechst 33258 in 0.15% Triton X-100 (0.5 mL) and incubated for 30 min at room temperature. Subsequently, they were observed under a fluorescence microscope at 20X magnification (EVOS FL digital inverted microscope, AMG).

### 3.8. Annexin V/PI Assay

The Annexin V-FITC/PI double staining assay was carried out according to the manufacturer’s instructions. Treated cells were collected into tubes, washed, re-suspended in 200 μL of PBS, and incubated with 5 μL of Annexin V and 5 μL of 20 μg/mL propidium iodide (PI) for 15 min at room temperature in the dark. Samples were washed with PBS and then analyzed by flow cytometer (15).

### 3.9. Western Blot Analysis

Western blot analysis was performed to determine the effect of compounds on COX-2 expression. Radioimmunoprecipitation assay (RIPA) buffer was used to lyse the cells, and the bicinchoninic acid (BCA) method was used to measure protein concentration. 20 µg of total protein was electrophoresed on a 12% sodium dodecyl-sulfate polyacrylamide gel electrophoresis (SDS-PAGE) and then transferred to a polyvinylidene difluoride (PVDF) membrane (Cat No. 162-017777; Bio-Rad Laboratories, CA, USA). Subsequently, the membranes were probed with anti-COX-2 (Cat No. ab179800, Abcam) and anti-β actin (used as an internal control) antibodies (Cat No. ab8227, Abcam), followed by goat anti-rabbit IgG H&L (HRP) (Cat No. ab6721; Abcam) as a secondary antibody. Chemiluminescence (ECL) was used to detect immunoreactive polypeptides. Results were quantified by densitometry scanning of the films using Image J software, version 1.8 (16).

### 3.10. Prostaglandin E2 Enzyme-Linked Immunosorbent Assay 

Prostaglandin E2 levels were measured according to the enzyme-linked immunosorbent assay (ELISA) kit instructions (R&D, Minneapolis, MN, USA). The absorbance at 450 nm was determined using a microplate reader (spectraMAX 340).

### 3.11. Statistical Analysis

The results represent the mean ± standard error of the mean from three different experiments. Statistical analysis was performed using a one-way analysis of variance (ANOVA) with Tukey's post hoc test, using GraphPad Prism version 8.0. P-values less than 0.05 were considered statistically significant.

## 4. Results and Discussion

### 4.1. Effect of Pyrrolic Compounds on PC12 Cell Viability 

**Figure 3.**
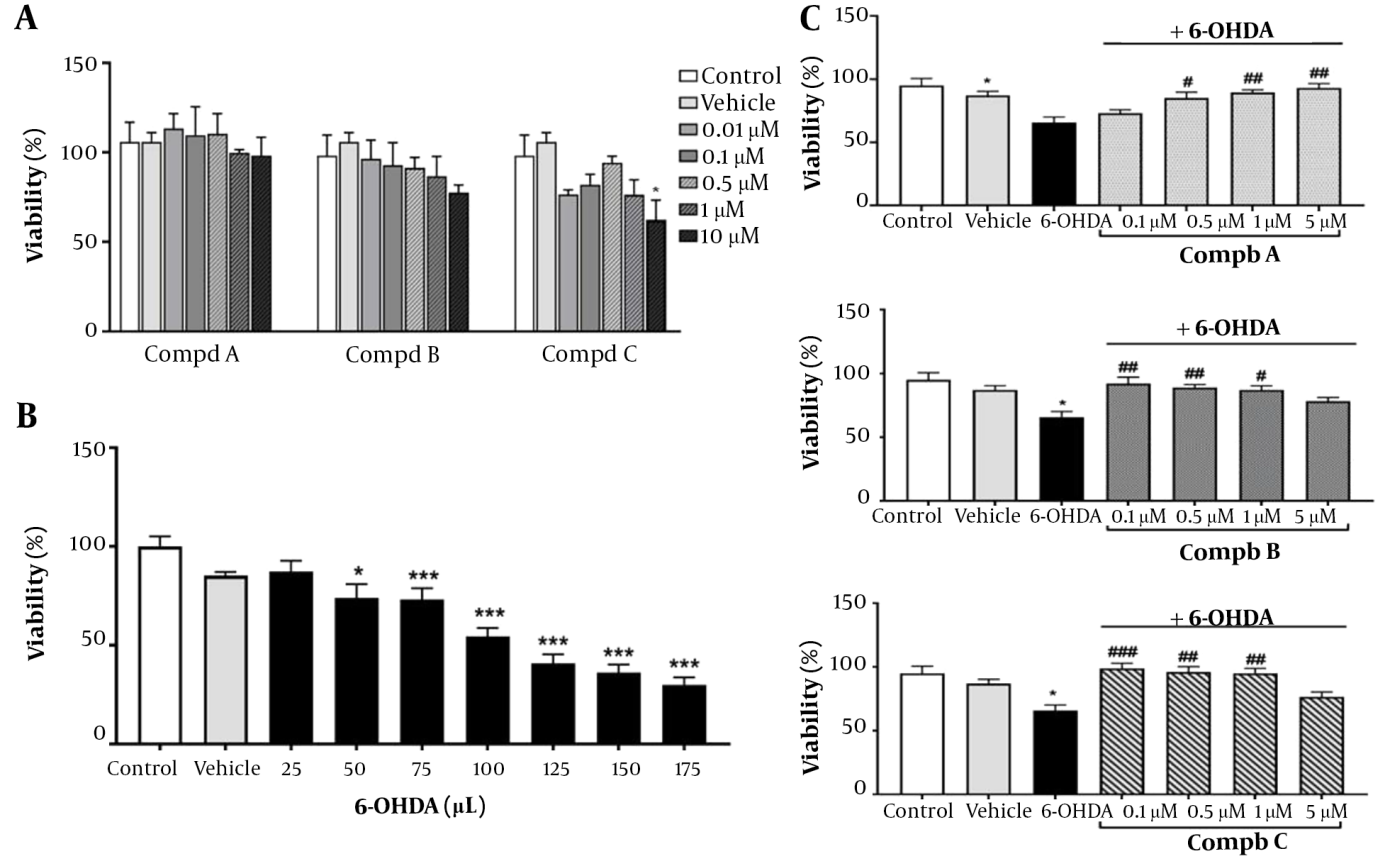
(A) Effect of pyrrole derivatives on cell viability. PC12 cells were treated with different concentrations of compounds A, B, and C for 24 h. (B) PC12 cells were exposed to 6-hydroxydopamine (6-OHDA;10, 25, 50, 75, and 100 μM) for 24 h. (C) Effect of pre-treatment with compounds A, B, and C (0.1, 0.5, 1, and 5 μM) on cell viability 24 h before 6-OHDA (100 μM) exposure. All data represent the mean ± SEM performed in at least 3 replicates. *P < 0.05 and ***P < 0.001 indicate a significant difference compared with the vehicle group. #P < 0.05, ##P < 0.01, ###P < 0.001 indicate a significant difference compared with the 6-OHDA group.

To assess the safety of pyrrolic compounds in our in vitro model, cells were treated with varying concentrations of pyrrolic compounds A, B, and C (0.01, 0.1, 0.5, 1, and 10 μM) for 24 h. Our results indicated that all 5 concentrations of the compounds did not significantly affect cell viability, except for 10 μM of compound C, which reduced PC12 cell viability to 62% (Figure 3A). Therefore, concentrations ranging from 0.1 to 5 μM of compounds A, B, and C were selected to assess their effects on 6-OHDA-induced toxicity (Figure 3C). 

Additionally, to determine the appropriate concentration of 6-OHDA for establishing the in vitro model of neurotoxicity, PC12 cells were exposed to 6-OHDA for 24 h at concentrations of 25, 50, 75, 100, 125, and 175 μM. 6-OHDA caused a significant decrease in PC12 cell viability in a concentration-dependent manner (Figure 3B). Since the concentration of 100 μM reduced the viability of PC12 cells by more than 50%, it was chosen to induce the neurotoxic model. 6-hydroxydopamine, known as oxidopamine, is a neurotoxic agent widely used to induce the degeneration of dopaminergic neurons in experimental models of PD (8). Neurodegeneration is induced by 6-OHDA through the production of free radicals, oxidative stress, inhibition of mitochondrial complexes I and IV (17), and activation of inflammatory responses (18).

The neuroprotective role of pyrrolic compounds was also evaluated by pretreating cells with compounds A, B, and C at four concentrations (0.1, 0.5, 1, and 5 μM) for 24 h, followed by 24 h exposure to 6-OHDA (100 μM). Compound A at concentrations of 0.5, 1, and 5 μM and compounds B and C at concentrations of 0.1, 0.5, and 1 μM reversed the cell cytotoxicity induced by 6-OHDA (Figure 3C). Accordingly, a concentration of 0.5 μM of compounds was selected for the following in vitro experiments. In line with these results, a previous study showed that new pyrrole-based hydrazide-hydrazones had a neuroprotective effect in an in vitro oxidative stress model induced by 6-OHDA in the SH-SY5Y cell line (19). Furthermore, another study by Kondeva-Burdina et al. confirmed that pyrrole-based hydrazones could protect isolated rat brain synaptosomes against toxicity induced by 6-OHDA (20). In another study, it was reported that pyrrole-2-carbaldehydes had a neuroprotective effect against oxygen-glucose deprivation/reperfusion injury in PC12 cells (21). Similarly, our observations revealed that pre-treatment with synthetic pyrrolic compounds protected PC12 cells against 6-OHDA-induced neurotoxicity.

### 4.2. Effect of Pyrrolic Compounds on 6-OHDA-Induced Lipid Peroxidation

The brain contains a large number of polyunsaturated fatty acids and is susceptible to oxidation (22). Lipid peroxidation is a complex process that occurs as a result of the interaction of oxygen-derived free radicals with polyunsaturated fatty acids, generating electrophilic aldehydes. Lipid peroxidation is used as a marker of oxidative stress and is highly evident in neurodegenerative diseases, including PD (23). Moreover, lipid peroxidation products alter the aggregation of α-synuclein, leading to the development of PD (24). Elevated levels of malondialdehyde (MDA), a product of lipid substrate oxidation, have been reported in the blood of advanced-stage PD patients (25).

**Figure 4.**
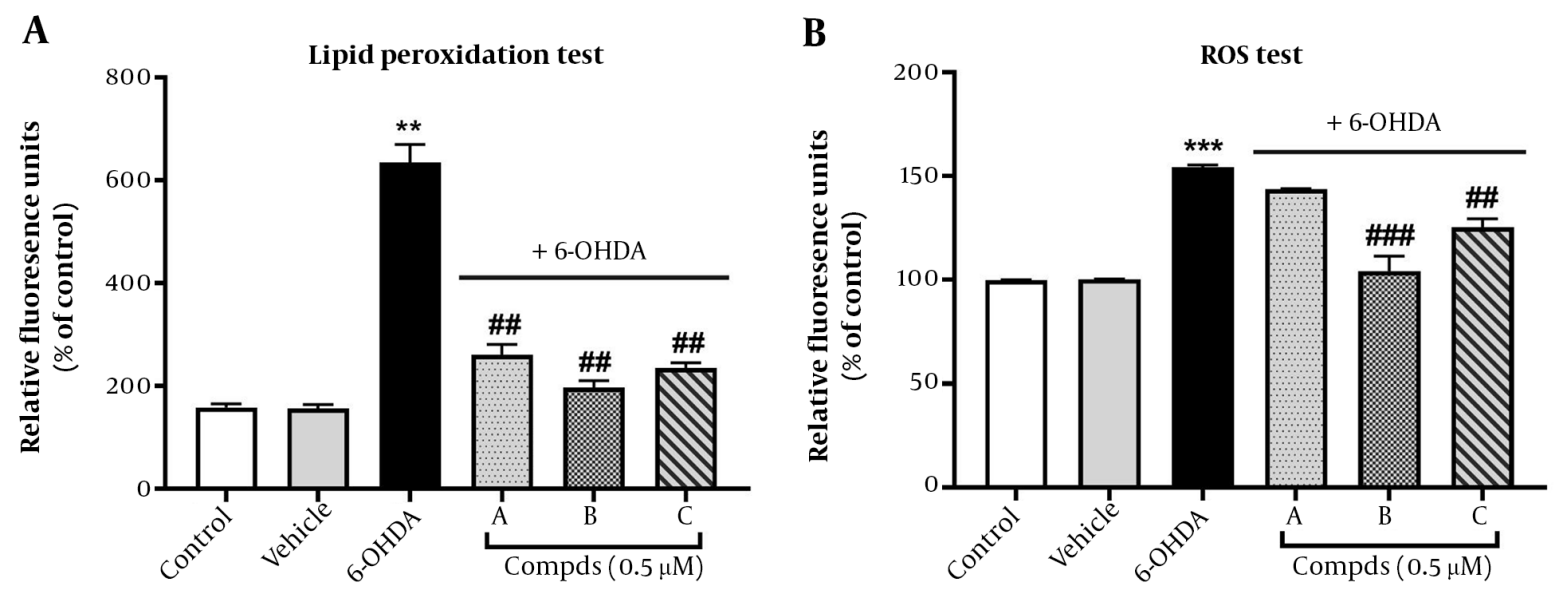
Effect of pre-treatment with compounds A, B, C on oxidative stress. Cells were pre-treated with compounds A, B, and C (0.5 μM) 24 h before 6-hydroxydopamine (6-OHDA; 100 μM) administration. (A) Lipid peroxidation and (B) reactive oxygen species generation were measured. Values shown are means ± SEMs of three independent experiments performed in four replicates. **P < 0.01 and ***P < 0.001 versus the untreated cells; ##P < 0.01 and ###P < 0.001 versus the 6-OHDA treated cells.

In this study, we assessed the protective role of pyrrolic compounds A, B, and C against lipid peroxidation using the TBARS assay. This assay measures MDA as a result of the oxidation of lipid substrates. Interestingly, elevated levels of MDA have been reported in the blood of advanced-stage PD patients (25). Our results indicated that 6-OHDA (100 μM) significantly induced lipid peroxidation in PC12 cells at 24 h, compared to untreated control cells. Lipid metabolism plays an important role in the progression of neurodegenerative diseases, and 6-OHDA is shown to be a suitable neurotoxin to induce a PD model for investigating lipid metabolism (26). Pre-treatment with pyrrolic compounds A, B, and C (0.5 μM) for 24 h before exposure to 6-OHDA remarkably reduced the percentage of lipid peroxidation, demonstrating the ability of these three pyrrolic compounds to prevent lipid peroxidation induced by 6-OHDA (Figure 4A). These findings support the protective role of pyrrolic compounds against oxidative stress in the PC12 cell model of PD.

### 4.3. Effect of Pyrrolic Compounds Pre-treatment on 6-OHDA-Induced Intracellular ROS Levels

The imbalance between ROS and antioxidant levels can lead to cell and tissue damage, including the destruction of dopamine neurons, which is ultimately the cause of PD (27). Neurons are continuously exposed to oxidative stress due to their high demand for ATP, resulting in the production of a high level of ROS from the mitochondrial electron transport chain (28). To investigate the involvement of ROS in 6-OHDA-induced neurotoxicity in PC-12 cells and determine whether the compounds influence neurotoxicity by modulating ROS levels, we measured the levels of intracellular ROS using the DCFH2-DA fluorescence staining assay. Our findings revealed that 6-OHDA (100 μM) significantly increased intracellular ROS levels compared to the control group after 24 h.

However, PC12 cells pre-treated with compounds B and C for 24 h exhibited lower levels of ROS compared to the 6-OHDA group. Compound A pre-treatment, on the other hand, did not significantly change the ROS levels (Figure 4B). 

Consistent with our results, the antioxidant activity of pyrrolic derivatives in the quinoline-2-carbaldehyde hydrazine structure has been demonstrated in CHO-K1 cells (29). Novel substituted formazans of 3,4-dimethyl-1H-pyrrole-2-carbohydrazide derivatives also exhibited in vitro antioxidant activity (30). Similarly, a reduced level of MDA has been reported previously for pyrrolic derivatives in inflamed rat tissue, confirming the antioxidant activity of these structures (31). Given that lipid peroxides can propagate ROS production (32), our results indicated the potential of synthetic compounds to inhibit lipid peroxidation and, subsequently, the generation of ROS induced by 6-OHDA. However, the inhibitory effect of compound A on ROS generation was not significant. These findings may be related to the characteristics of substituents, such as whether they are electron-donating or accepting groups or even the position of the substituents in the aniline derivatives.

### 4.4. Effects of Pyrrolic Compounds on the Nuclear Morphological Changes Induced by 6-Hydroxydopamine

**Figure 5.**
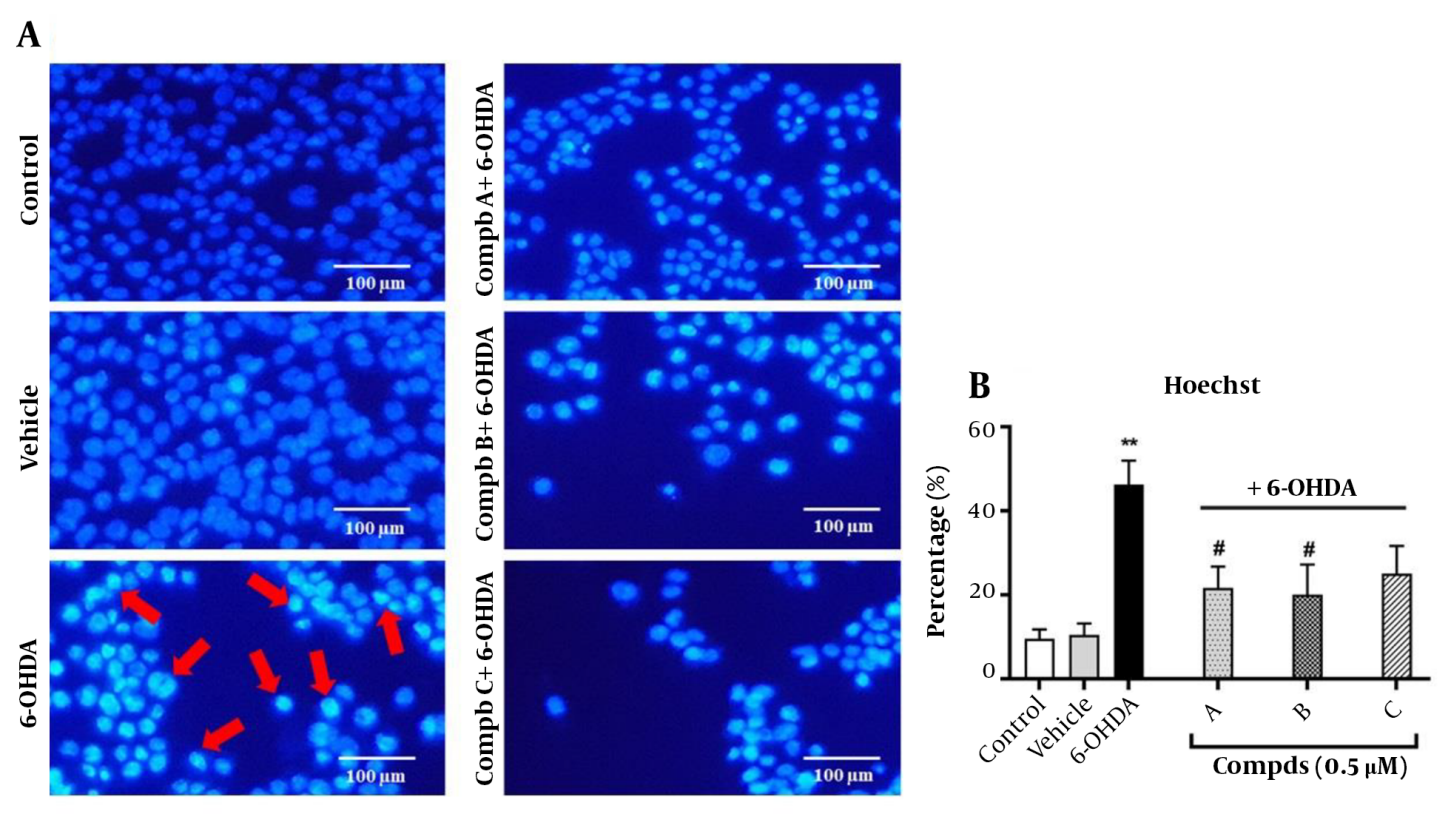
Effect of compounds A, B, and C (0.5 µM) on 6-hydroxydopamine (6-OHDA)-induced apoptosis in PC12 cells. Cells were stained with the DNA-binding fluorochrome Hoechst 33258. (A) Red arrows represent apoptotic cells. (B) Histograms show the ratio of condensed nuclei to total nuclei. **P < 0.01 versus the untreated cells; #P < 0.05 versus the 6-OHDA treated cells. The morphological changes were observed using a fluorescent microscope (10X).

To verify that cell apoptosis is mediated through lipid peroxidation, we assessed apoptosis induction in the cells treated with 6-OHDA and pre-treated with compounds A, B, and C. One approach to demonstrate apoptosis induction is evaluating nuclear morphological alterations following treatment with 6-OHDA and pre-treatment with the compounds. For this purpose, Hoechst 33258 staining was used. Our findings indicated that the nuclei of control cells had rounded and homogeneous features without abnormalities, whereas the nuclei of cells exposed to 100 μM of 6-OHDA for 24 h were condensed and exhibited highly bright fluorescence, which confirms apoptosis induction. Interestingly, the changes observed upon 6-OHDA treatment were significantly prevented following pre-treatment of cells with compounds A and B. However, compound C did not affect the nuclear morphology, and the number of condensed and bright nuclei remained unchanged compared to the 6-OHDA group (Figure 5). 

### 4.5. Effects of Pyrrolic Compounds on Apoptosis in PC12 Cells Upon Pre-treatment with the Compounds

For further validation of the protective role of pyrrolic compounds in apoptosis induction, flow cytometric analysis was performed using an Annexin/PI kit. In this assay, Annexin V fluorescence indicates the number of apoptotic cells, while PI shows necrotic cells. Annexin variable fluorescence optical system (V-FLUOS)/PI results are shown in Table 1. The percentage of early apoptotic cells increased from 0.81% in the control cells to 15.15% in the cells exposed to 6-OHDA for 24 h. The percentage of early apoptotic cell population pre-treated with compounds A and B significantly decreased compared to the 6-OHDA group, while compound C did not significantly reduce the percentage of early apoptotic cells. Furthermore, the percentages of late apoptotic cells were 5.33% and 31.95% in the control and 6-OHDA-treated cells, respectively. When cells were pre-treated with compounds A, B, and C, the population of late apoptotic cells decreased, although the differences were not statistically significant. These results are in agreement with the Hoechst staining test and support the inhibitory role of compounds A and B in apoptosis induced by 6-OHDA.

**Table 1.** Effects of Pyrrolic Compounds on Apoptosis Induced by 6-OHDA in PC12 Cells ^a,b^

Groups	Vital Cells (%) An-/PI-	Early Apoptosis (%) An+/PI-	Late Apoptosis (%) An+/PI+	Necrosis (%) An-/PI+
**Control**	83.65 ± 9.75	0.81 ± 0.34	5.33 ± 2.00	1.66 ± 0.46
**Vehicle**	88.35 ± 4.95	1.23 ± 0.26	5.39 ± 0.95	1.53 ± 0.23
**6-OHDA**	49.00 ± 17.4	15.15 ± 1.55 ^c^	31.95 ± 14.15	3.87 ± 1.77
**Compd A + 6-OHDA**	70.55 ± 3.45	6.79 ± 2.19 ^d^	20.90 ± 5.1	1.78 ± 0.58
**Compd B + 6-OHDA**	70.55 ± 4.45	6.42 ± 1.34 ^d^	17.45 ± 3.25	5.54 ± 0.11
**Compd C + 6-OHDA**	67.85 ± 2.15	12.7 ± 1.5	15.80 ± 0.20	3.80 ± 1.03

^a^ The data presented are the mean ± SEM of 3 independent experiments.

^b^ PC12 cells were also pre-treated with 0.5 µM of compounds A, B, and C for 24h.

^c^ P < 0.01 versus the untreated cells.

^d^ P < 0.05 versus the 6-OHDA treated cells.

Based on these data, we suggest that pre-treatment with compounds A, B, and somehow C protects PC12 cells from lipid peroxidation and apoptosis induction by 6-OHDA treatment in the in vitro model.

### 4.6. Effects of Pyrrolic Compounds on Alterations in Cyclooxygenase-2 Protein Expression Following Pre-treatment with the Compounds in 6-Hydroxydopamine-Treated PC12 Cells

Increased expression of COX-2, as well as elevated PGE2 levels in the brain, are associated with inflammation and neurodegenerative diseases such as PD (33). Interestingly, a study by Kumagai et al. demonstrated a correlation between COX-2 expression and the accumulation of lipid peroxidation products in macrophage cells (34). To investigate this association in our study, Western blotting analysis was employed to determine COX-2 expression.

Our results indicated that COX-2 expression in cells treated with 6-OHDA significantly increased compared to the control group, which is in line with previous studies showing that 6-OHDA treatment induces COX-2 activation in neuronal cell lines such as Neuro-2a and SH-SY5Y, leading to elevated PGE2 secretion and the induction of a pro-inflammatory cascade (35).

**Figure 6.**
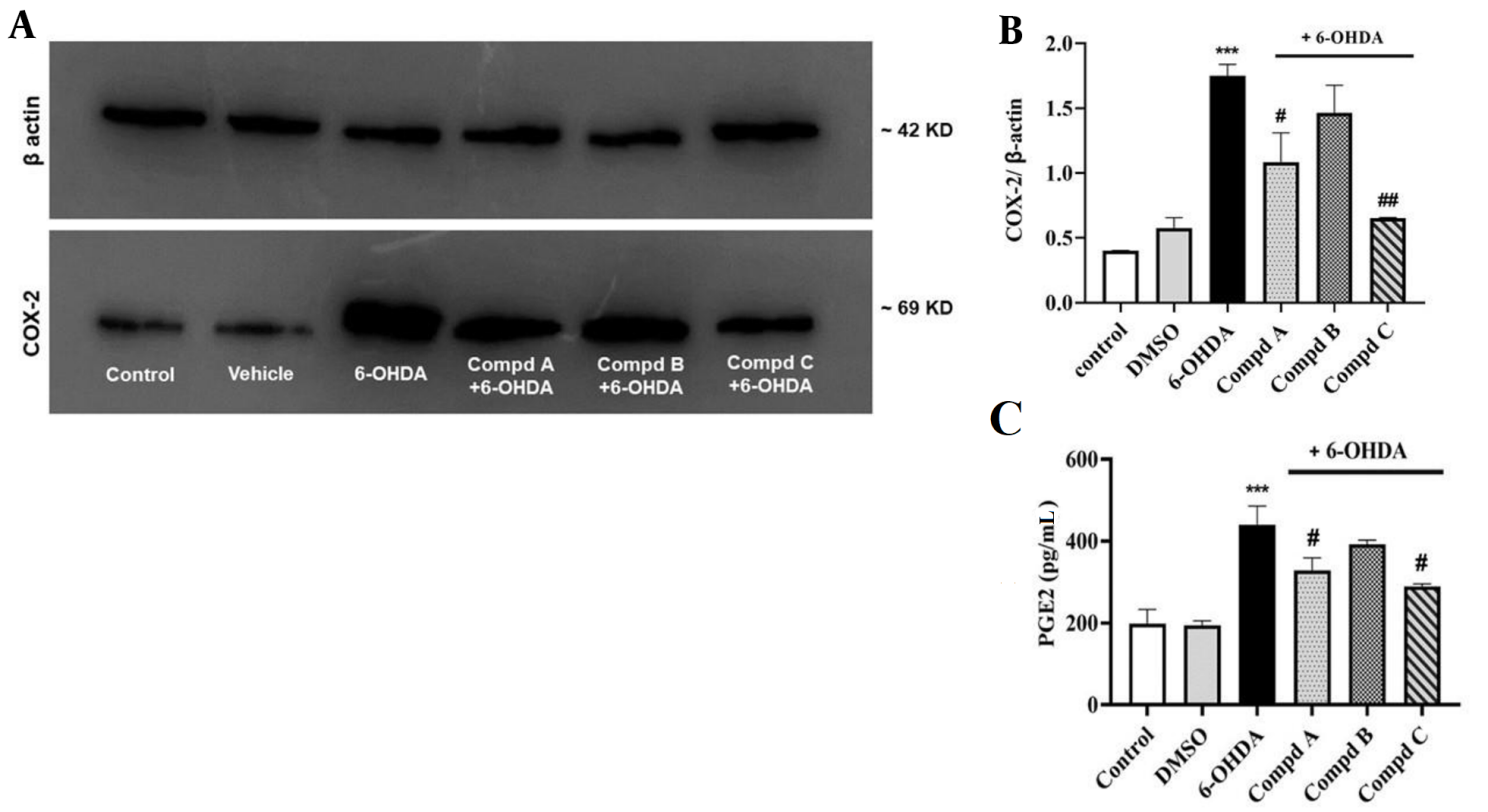
Effect of compounds A, B, C (0.5 µM) on cyclooxygenase-2 (COX-2) protein expression and PGE2 level induced by 6-hydroxydopamine (6-OHDA) in PC12 cells. (A) The relative level of COX-2 expression was calculated and normalized to the loading control. (B) Corresponding protein levels were assessed using densitometry. (C) Effect of compounds A, B, and C (0.5 µM) on PGE2 levels in 6-OHDA-treated PC12 cells. Values shown are means ± SEMs of 3 independent experiments performed in 3 replicates. *** P < 0.001 versus the untreated cells; # P < 0.05 and ## P < 0.01 versus the 6-OHDA treated cells.

Additionally, the present data uncovered that pre-treatment with compounds A and C, 24 h before exposure to 6-OHDA, prevented the enhancement of COX-2 expression induced by 6-OHDA. Compound B also decreased COX-2 expression; however, it was not statistically significant (Figure 6A). 

Our literature survey revealed that inhibiting COX-2 activity in microglial cells can suppress progressive degeneration in the retrograde lesion model of PD induced by 6-OHDA in rats (36). Furthermore, various studies have highlighted the anti-inflammatory role of pyrrole-based compounds by inhibiting COX activity (11, 37). In this regard, it has been reported that the 1,5-diaryl pyrrole scaffold has a high affinity for the COX-2 active site with the ability to inhibit COX-2 activity (38). Additionally, 1,5-diarylpyrrol-3-sulfur derivatives have been shown to possess strong inhibitory efficiency against COX-2, along with anti-inflammatory and anti-nociceptive effects in in vivo models (39). In agreement with these findings, our results demonstrated that all 3 pyrrolic derivatives could inhibit COX-2 expression induced by 6-OHDA, although this inhibition by compound B was not remarkable. Indeed, it is likely that different substituents of pyrrole compounds alter the electrostatic and hydrophobic characteristics of the aromatic ring, which in turn affects the lipophilicity and the amount of the compound taken up by the cells.

### 4.7. Effects of Pyrrolic Compounds on Prostaglandin E2 Levels Following Pre-treatment with Compounds in 6-Hydroxydopamine-Treated PC12 Cells

Prostaglandin E2 plays a key role in inducing the expression of a cassette of pro-inflammatory genes and promoting neurodegeneration in many neurodegenerative diseases (40, 41). Prostaglandin E2 can also enhance microglia-mediated α-synuclein aggregation in the mouse 1-methyl-4-phenyl-1,2,3,6-tetrahydropyridine (MPTP) model of PD (42). In addition, PGE2 is considered a hallmark of increased COX-2 activity (43). Thus, the findings of the PGE2 assay will help us further verify our outcomes.

In this context, we found that 6-OHDA significantly amplified PGE2 levels, while pre-treatment with compounds A and C, 24 h before 6-OHDA administration, reduced PGE2 levels. However, compound B had no significant effect on PGE2 generation in the 6-OHDA treated cells (Figure 6C). These results suggest that both compounds A and C might act by suppressing the COX-2/PGE2 pathway, which is not attributed to compound B.

## 5. Conclusions

Novel synthetic pyrrole derivatives can prevent 6-OHDA-induced neurotoxicity in PC12 cells. It appears that the mechanism of action of these pyrrolic compounds involves the suppression of COX-2 expression, a reduction in PGE2 levels, and the inhibition of ROS production, lipid peroxidation, and oxidative stress-induced apoptosis. These actions ultimately result in a neuroprotective effect against 6-OHDA. However, the complete molecular milieu that links all these events together still needs to be elucidated.
